# *Paecilomyces lilacinus *causing debilitating sinusitis in an immunocompetent patient: a case report

**DOI:** 10.1186/1752-1947-6-86

**Published:** 2012-03-26

**Authors:** Gentle Wong, Robert Nash, Kushal Barai, Raksha Rathod, Arvind Singh

**Affiliations:** 1 Department of Otolaryngology, Northwick Park Hospital, Middlesex, HA1 3UJ, UK; 2 Department of Microbiology, Northwick Park Hospital, Middlesex, HA1 3UJ, UK

## Abstract

**Introduction:**

Since the discovery of the first documented case of *Paecilomyces *in 1963, only five cases of *Paecilomyces *sinusitis have been described to date and all of them have predisposing factors such as immunocompromised status or prior nasal surgery. We present the first case of *Paecilomyces lilacinus *sinusitis in a fit young woman with no identified predisposing factors. To the best of our knowledge, this is the first known case in the UK and in Europe.

**Case presentation:**

A 20-year-old Iraqi woman who has lived in the UK for the past five years presented with rhinorrhea, hyposmia, and nasal obstruction. She was previously fit and well and had no significant medical history. Imaging revealed a fungal infection that was eventually revealed on cytological examination to be *P. lilacinus*.

**Conclusions:**

*P. lilacinus *is both a difficult and important organism to identify because it has intrinsic anti-fungal resistance. In our case, the infection was severe and recurrent, and the organism demonstrated resistance to common oral anti-fungal agents. There was a delay in its diagnosis, owing to its similarity in appearance to *Penicillium *and a difficulty in distinguishing between the two without specialized knowledge of fungal taxonomy. In the field of otolaryngology, *Paecilomyces *is relatively unknown. Our intention is to raise awareness of this organism as well as to describe the challenges in its management.

## Introduction

Fungal sinusitis is a common infection and its prevalence has increased significantly in the past 30 years [1]. This trend could be attributed to modern immunosuppressive agents, overuse of antibiotics, and increased public awareness. Generally speaking, fungal sinusitis can be classified as invasive - determined by the presence of fungal hyphae within the mucosa, submucosa, bone, or blood vessels of the paranasal sinuses - or non-invasive. Species of *Aspergillus *are the most common agents in fungal sinusitis and the next most common are a vast array of fungi, including *Candida *spp., *Cryptococcus neoformans*, *Bipolaris*, and zygomycetes. Effective management depends on the type of sinusitis as well as the causative fungus/fungi and can range from aggressive surgical debridement and systemic anti-fungal therapy (as in most cases of acute/chronic invasive fungal sinusitis) to surgical removal of the fungal source and long-term use of topical nasal steroids (in cases of allergic fungal sinusitis). Occasionally, unusual organisms are detected and these may have implications for successful eradication and treatment.

*Paecilomyces lilacinus *is an opportunistic fungus commonly found in the soil but is seldom pathogenic for humans. Only five cases of a fungal sinusitis caused by *P. lilacinus *have been documented [2-6], and all five patients were associated with either impaired host defenses or prior surgical procedures. Here, we report the first case of *P. lilacinus *fungal sinusitis in the UK and Europe and briefly review the literature on *Paecilomyces *infections.

## Case presentation

**Figure 1:**
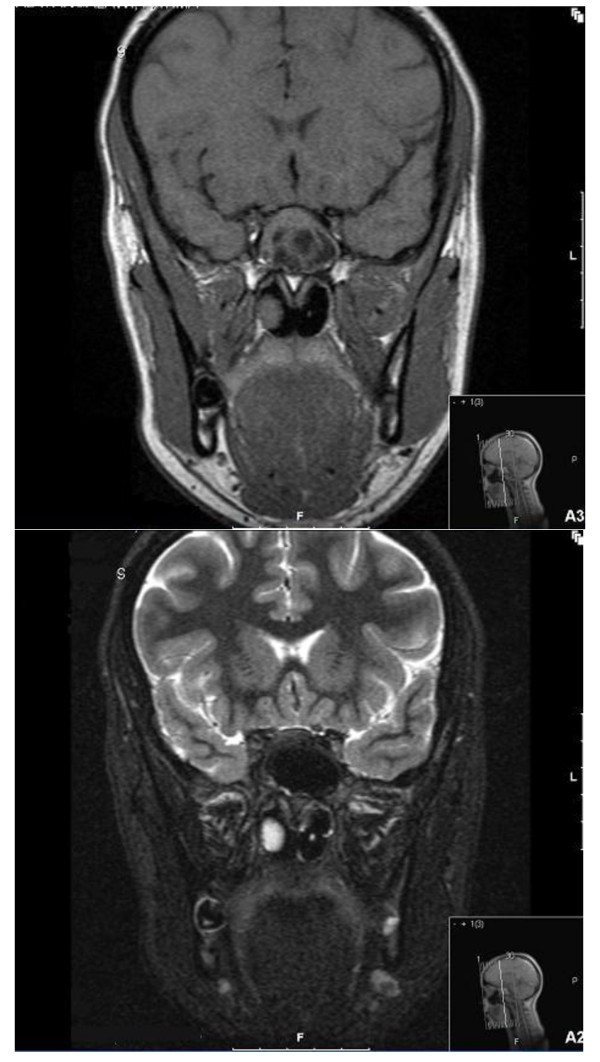
**Magnetic resonance images of sinuses demonstrate a fungal infection**. Coronal T1-weighted (above) and T2-weighted (below) images demonstrate enlargement of the left sphenoid sinus. The posterior aspect of the left nasal polyp and the contents of the large sphenoid sinus are of low T2 and short inversion time inversion recovery (STIR) signal intensity, indicating the presence of coexisting fungal infection.

A 20-year-old Iraqi woman who has lived in the UK for the past five years presented with rhinorrhea, hyposmia, and nasal obstruction. A computed tomography scan of her sinuses revealed a left-sided posterior nasal polyp associated with a sphenoidal mucocele. On both T1- and T2-weighted images, magnetic resonance imaging (MRI) of the sinuses demonstrated hypointensity in keeping with fungal infection (Figure 1). She underwent endoscopic sinus surgery and clearance of the collection. A microbiology specimen isolated *Staphylococcus aureus*. Histology revealed numerous fungal hyphae.

**Figure 2:**
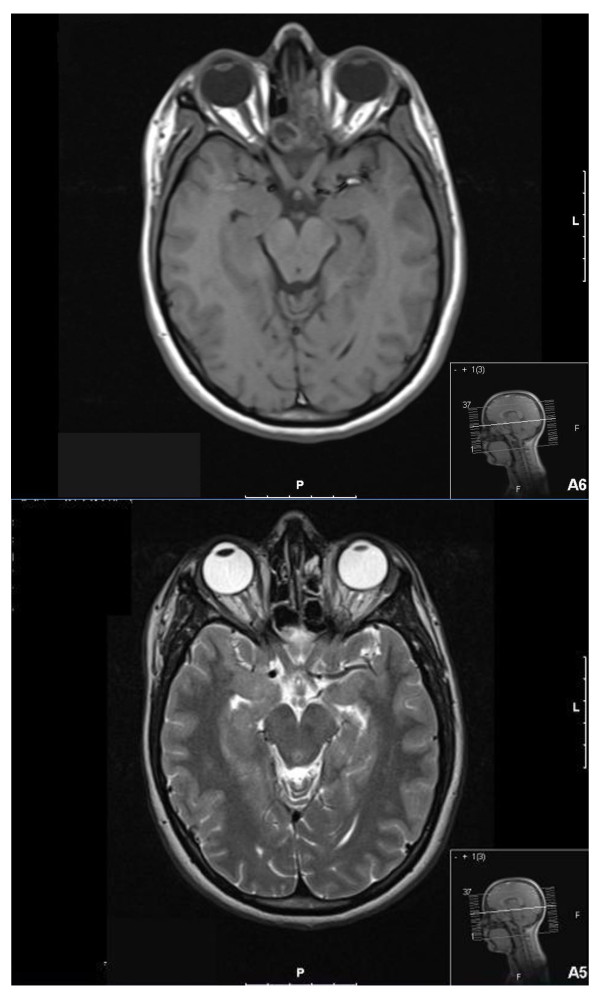
**Magnetic resonance images of sinuses demonstrate a recurrence of the fungal infection**. Axial T1-weighted and T2-weighted images show opacification of the sphenoid sinus on the left with signal characteristics of fungal infection. The infection extends into the left posterior and anterior ethmoidal air cells.

**Figure 3:**
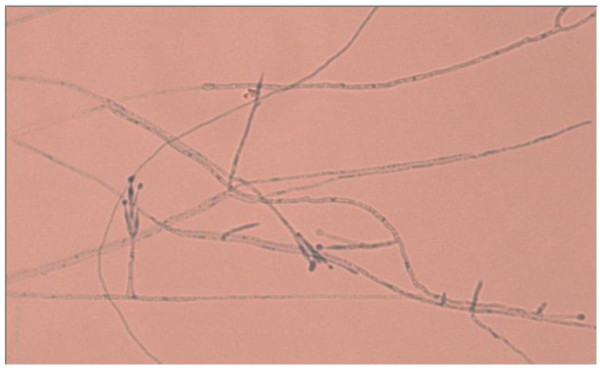
**Septate hyaline hyphae, conidiophores, phialides, conidia, and chlamydospores**. Conidiophores (3 to 4 μm wide and 400 to 600 μm long) are branched and carry the phialides at their tips. The phialides are swollen at their bases and taper toward their apices.

After the operation, her symptoms initially improved but subsequently recurred despite topical steroid and saline douches. She continued to have headaches and nasal discharge. Repeat MRI described a more extensive infection, extending into the ethmoidal air cells (Figure 2). During revision surgery, offensive florid fungal sinusitis was extensively cleared. Cytology identified *P. lilacinus *(Figure 3). She made a good post-operative recovery.

## Discussion

A MEDLINE search for all cases of *Paecilomyces *infections involving paranasal sinuses was performed to assess frequency, clinical presentation, and treatment (Table 1). We found that all five patients who have contracted the infection were associated with either impaired host defenses or previous nasal procedures.

**Table 1:** *Paecilomyces *paranasal sinus infections

Case	Year	Reference	Country	Age in years/Gender	Predisposing factors	Treatment
1	1980	[2]	USA	47/Female	Previous nasoantrostomy	Surgery

2	1982	[3]	USA	47/Female	Previous nasoantrostomy	Surgery

3	1996	[4]	USA	22/Female	Myeloid leukemia	AMB, 5FC, and ITZ

4	1997	[5]	USA	57/Female	Diabetes mellitus	Surgery, AMB, and ITZ

5	2000	[6]	India	8/Male	Previous nasoantrostomy	Surgery and ITZ

5FC: 5-flucytosine; AMB: amphotericin B; ITZ: itraconazole.

In all parts of the body, there have been only four published reports of a cytologically confirmed infection due to *P. lilacinus *in a patient without predisposing factors [7-10]. Long-term treatment with itraconazole led to resolution of the ocular and cutaneous infections [7,9].

The two major pathogenic species of genus *Paecilomyces *are *P. lilacinus *and *P. variotti. P. lilacinus *is documented as the more pathogenic agent while also being more resistant to anti-fungal therapy, notably amphotericin B and flucytosine [11,12]. This underscores the need to accurately identify the correct species. Worryingly, our specimen was also resistant to itraconazole, which was the successful anti-fungal agent in previous cases. Our specimen was sensitive to caspofungin and voriconazole, both requiring parenteral administration in our hospital.

Furthermore, in our case, morphological identification using standard methods was inconclusive. The sample was inoculated onto sabouraud dextrose agar + chloramphenicol and sabouraud dextrose agar + chloramphenicol + actidione. The plates were incubated at 37°C and 30°C for 10 days. Growth was observed at 30°C on both plates at days three to five, and eventually the isolate grew at 37°C after further incubation. The cultures grew as a white mold that failed to pigment initially, but after several weeks of further incubation, the culture became lilac. This explains the difficulty in initial identification. Ultimately, a definitive identification was established through molecular analysis at the Mycological Reference Laboratory in Bristol, UK.

The problem with identification has been reported before, as micro-morphological analysis reveals that the reproductive structures of *Paecilomyces *and *Penicillium *are similar in appearance and difficult to distinguish without specialized optics and measuring devices not available in most clinical laboratories. Gottlieb and Atkins [9] showed that the internal transcribed spacer (ITS) regions within the recombinant deoxyribonucleic acid (rDNA) complex were effective molecular targets for the identification of *Paecilomyces*. The difficulty in isolating the organism suggests that, in some cases, sinusitis could be caused by *P. lilacinus *but attributed to other microorganisms, as occurred in our case, in which *S. Aureus *was initially identified as the causative agent, even though hyphae were also present.

## Conclusions

We report the first case of *P. lilacinus *sinusitis in an immunocompetent adult who had had no prior nasal surgery. Incidentally, this is also the first case in the UK and in Europe. *P. lilacinus *may cause aggressive infections and often demonstrates multi-drug resistance. In our case, these drugs included itraconazole, fluconazole, amphotericin, and flucytosine. This is therefore an important new differential diagnosis to consider because of the organism's potential multi-anti-fungal resistance, which can complicate medical management, and the difficulty in its identification, which can lead to delays in initiating treatment.

## Consent

Written informed consent was obtained from the patient for publication of this case report and accompanying images. A copy of the written consent is available for review by the Editor-in-Chief of this journal.

## Abbreviations

MRI: magnetic resonance imaging.

## Competing interests

The authors declare that they have no competing interests.

## Authors' contributions

GW was the chief author of the manuscript. RN was involved in revising the manuscript critically for important intellectual content. KB was involved in the patient's care and the acquisition of the imaging data. He also obtained the patient's consent for publication of this case report and accompanying images. RR was the specialist biomedical scientist who examined and identified the organism. AS was the consultant who supervised the case report. All authors read and approved the final manuscript.
